# Development of Real-Time Hand Gesture Recognition for Tabletop Holographic Display Interaction Using Azure Kinect

**DOI:** 10.3390/s20164566

**Published:** 2020-08-14

**Authors:** Chanhwi Lee, Jaehan Kim, Seoungbae Cho, Jinwoong Kim, Jisang Yoo, Soonchul Kwon

**Affiliations:** 1 Department of Electronic Engineering, Kwangwoon University, Seoul 01897, Korea; chanheui0102@gmail.com (C.L.); jsyoo@kw.ac.kr (J.Y.); 2 Electronics and Telecommunications Research Institute (ETRI), Daejeon 34129, Korea; kimjhan@etri.re.kr (J.K.); csb60237@etri.re.kr (S.C.); jwkim@etri.re.kr (J.K.); 3 Graduate School of Smart Convergence, Kwangwoon University, Seoul 01897, Korea

**Keywords:** azure kinect, deep-learning, gesture interaction, hand detection, hologram display

## Abstract

The use of human gesturing to interact with devices such as computers or smartphones has presented several problems. This form of interaction relies on gesture interaction technology such as Leap Motion from Leap Motion, Inc, which enables humans to use hand gestures to interact with a computer. The technology has excellent hand detection performance, and even allows simple games to be played using gestures. Another example is the contactless use of a smartphone to take a photograph by simply folding and opening the palm. Research on interaction with other devices via hand gestures is in progress. Similarly, studies on the creation of a hologram display from objects that actually exist are also underway. We propose a hand gesture recognition system that can control the Tabletop holographic display based on an actual object. The depth image obtained using the latest Time-of-Flight based depth camera Azure Kinect is used to obtain information about the hand and hand joints by using the deep-learning model CrossInfoNet. Using this information, we developed a real time system that defines and recognizes gestures indicating left, right, up, and down basic rotation, and zoom in, zoom out, and continuous rotation to the left and right.

## 1. Introduction

Gesture interaction technology that measures and analyzes the movement of the user’s body to control information devices or to link with content has been the topic of many studies [1,2,3,4,5,6,7]. Among them, the hand is the most easily used and is a medium capable of various formations owing to its high degree of freedom. Therefore, most gesture interactions include gestures that involve the use of the hands. Human interaction with a computer by way of gesturing relies on gesture interaction technology, representative examples of which are Azure Kinect and Leap Motion from Leap Motion, Inc. This technology enables gesture interaction to be used to easily control a variety of devices.

Recently, gesture recognition using a sensor such as Azure Kinect has been applied to a wide range of fields from smart home applications, medical applications, to automotive applications [8,9,10,11,12,13]. Through the gesture interaction of the smart home, household appliances are available without touching them directly. In medical applications, gesture interaction helps remote remedial exercise of burn patients to rehabilitate. In the automotive field, it is difficult for a driver to use a touch screen while driving. Gesture interaction allows drivers to control touch screen operations with gestures.

Tabletop holographic display is a device that allows the viewer to observe 3D hologram contents created from various angles with multiple cameras and view them anywhere from 360 degrees [14,15,16]. The system allows multiple viewers to view digital hologram images in the horizontal 360-degree direction by using digital micromirror device (DMD) capable of high-speed operation as a spatial light modulator (SLM). In addition, by applying a lenticular lens, holographic images can be viewed in a range of 15 degrees or more in the vertical direction. The device can display 22,000 binary hologram image data per second, with 1024 and 768 pixels in the horizontal and vertical directions, respectively.

A color hologram display device consisting of a total reflection prism illuminates the DMD and a prism capable of separating and recombining the three primary colors of light by applying three DMDs, a laser source emitting red, green and blue laser light, and the use of a fiber-based laser combination of the wavelengths 660, 532, and 473 nm. Because the holographic image is created by the interference of light, the hologram resulting from the tabletop holographic display must be observed in a dark environment from which all light is excluded. Therefore, in a dark environment without light, the display needs to be controlled by a computer to control the image displayed on the tabletop holographic display.

**Figure 1:**
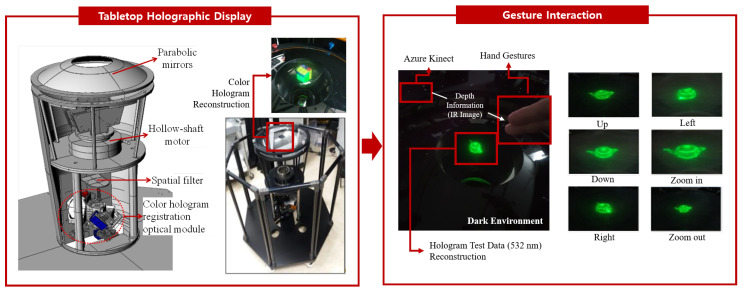
Overview of the gesture recognition for the tabletop holographic display.

Since light is not present in the experimental environment, only depth information is used to develop gesture interaction using hand gestures. Thus, high accuracy hand detection using color information is not applicable. In this experiment, we use only the depth image with the high quality depth camera Azure Kinect. In addition, depth images are disturbed by structures such as optical components and eye-tracking cameras attached to tabletop holographic displays. To interact with the tabletop hologram display, we designed a new pipeline as shown in Figure 1. This pipeline is described in full in the next sections, beginning with the physical setup, to our proposed method for gesture interaction.

## 2. Background Theory

### 2.1. Azure Kinect

**Figure 2:**
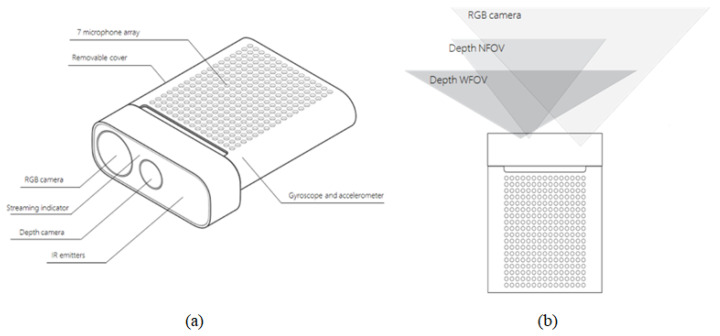
Azure Kinect. (**a**) Azure Kinect configuration; (**b**) Azure Kinect view field. (source: www.docs.microsoft.com)

3D depth recognition technology consists of stereo-type, structured light, and Time-of-Flight (ToF). Stereo-type uses a viewpoint mismatch between two 2D cameras, similar to the principle that a person measures distance using both eyes. The structured light method recognizes the depth by calculating the amount of change in a pattern by scanning a specific light pattern on an object. The ToF method recognizes depth by calculating the travel time of light reflected from an object. The ToF method can acquire a better depth quality than other methods in an indoor environment. Azure Kinect is a Microsoft’s ToF-based depth camera released in 2019. Figure 2a shows the configuration of Azure Kinect. Azure Kinect provides high quality depth information. Figure 2b shows the Azure Kinect view field. Depth Narrow-Field of view (NFOV) is a mode that provides depth information of a narrow area, and Depth Wide-Field of view (WFOV) is a mode that provides depth information of a wide area.

### 2.2. Gesture Interaction

Gesture interaction takes place when a user makes a gesture to command a device such as a computer. In other words, it entails noncontact interaction between the user and the computer. Among the human limbs, the hand is the most easily used and is a medium capable of conveying various expressions; thus, gesture interaction mostly occurs by using hand gesture interaction, which requires the use of the hands. Hand gesture interaction necessitates accurate hand detection and hand joint information [17,18].

**Figure 3:**
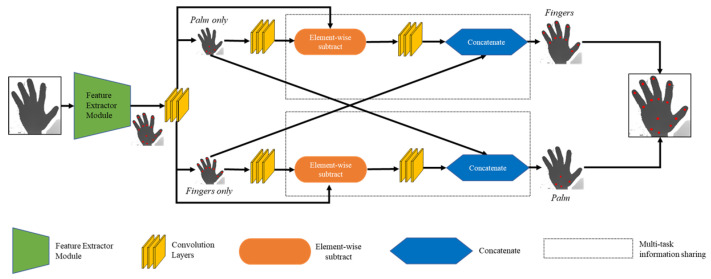
CrossInfoNet structure.

CrossInfoNet [19] is a deep learning model that detects hands using depth information. Figure 3 shows the structure of CrossInfoNet. Unlike other existing models that detect the entire hand image to find joint information [20,21,22,23,24,25], CrossInfoNet detects the entire hand once, and then redetects the palm and fingers, respectively. First, the entire hand is detected to obtain approximate finger and palm joint information. The acquired palm and finger joint information is re-extracted from each of the two different branches. The joint information that is found again is transferred to the other branch. In other words, the information about the palm of the hand is delivered to the branch where the finger is found, and the information about the finger is transferred to the branch on which the palm is found. The branch containing the palm information also contains rough palm joint information received via a skip connection together with details of fine finger information shared with the palm joint information. When these types of information are subtracted, the coarse palm information and the fine palm information disappear, and only the fine finger information remains. In this way, finger joint information is obtained. In addition, in the branch in which the finger is found precisely, subtracting is performed using the approximate finger joint information received via the skip connection, the finger joint information found finely, and the sophisticated palm information shared. The result is elaborate palm information. Finger and palm information obtained by using this process is continuously shared with each finding as learning progresses, resulting in more accurate finger and palm joint information. The joint information of the last finger and palm joint is acquired to obtain the joint information of the entire hand. When hand detection and hand joint information are obtained, it is defined using the hand joint information obtained by the user.

It is important that each gesture should be defined such that it is intuitive and easy for the user to learn [26]. As a good example, operating a smartphone is similar to the operations performed when handling a book or paper in real life, such as switching the screen by swiping to the next page or pushing and lowering it, thus users are easily able to use the phone without the need to learn. Gesture interaction allows users to control the device they intend using without having to touch the mouse, keyboard, remote control, or screen. In addition, people with disabilities can control the device with simple hand gestures, thereby improving usability and convenience. In addition, gesture interaction is useful in situations in which it is difficult to operate other devices, such as a doctor working in an operating room while wearing work gloves.

## 3. Proposed Method

### 3.1. System Configuration

**Figure 4:**
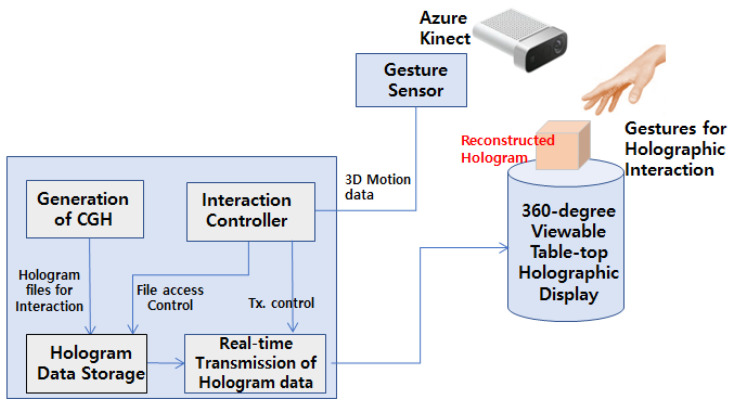
Block diagram of real-time interactive holographic display by hand gestures.

When the gesture determined in the video is input to the server using UDP/IP socket communication, the server transmits the corresponding hologram to the sending end. The hologram reads the received signal and shows the hologram display corresponding to the signal. The 3D hologram data created via computer-generated holography (CGH) are stored in the hologram data storage section. The information of the user’s hand gesture obtained from the Azure Kinect video is transferred to the Interaction Controller section using UDP/IP socket communication. In the Interaction Controller section, the hologram sending unit requests new hologram contents that have undergone appropriate actions for the corresponding gesture. The hologram transmitter that receives the request displays the hologram image with the gesture on the 360-degree Viewable Tabletop Holographic Display. The user can observe the new hologram display by viewing the gesture action taken in real time. Figure 4 shows the overall structure of the 3D holographic display system capable of interacting with hand gestures.

### 3.2. Proposed Gesture Interaction

**Figure 5:**
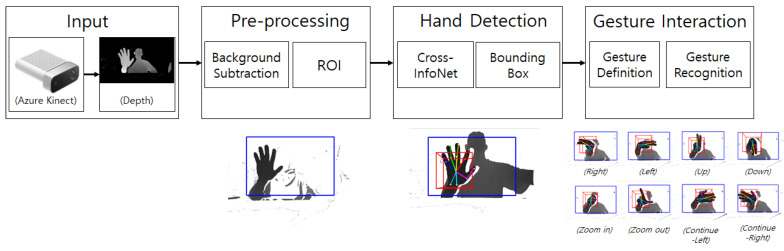
Framework of the hand gesture recognition system based on depth frames.

Color information is used for most high performance hand detection models [27]. However, color information cannot be used in the tabletop holographic display environment. Therefore, using only depth information, the characteristics of the tabletop holographic display installed in many other structures are taken into account. Then, the necessary depth information is retrieved using background subtraction and Region of Interest (ROI) to detect the hand. The ROI is the area limited to processing only the region of interest in the image. Furthermore, gestures are defined using the joint information of the detected hand. Then, when a gesture corresponding to the subject’s motion is detected, the gesture prediction is output in real time. The system was designed to operate with a delay of one second between gestures. Figure 5 shows the framework of the hand gesture recognition system based on depth frames.

#### 3.2.1. Background Subtraction

**Figure 6:**
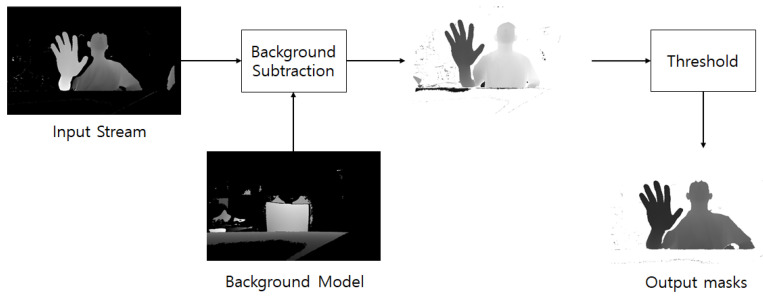
Background subtraction.

We use the depth difference between the first frame and the next frame when the image is turned on, and we used a threshold to obtain the depth information of the image when it exceeds the threshold. Figure 6 shows the background subtraction method. When the camera is turned on, only the background or structures other than the user are present on the screen in the first frame that is received. Thereafter, the user exists in the incoming frames, and the depth difference is continuously calculated from the first frame. The background and surrounding structures are erased using only the depth information of the image with a depth difference value exceeding a predetermined threshold, it becomes the image in which only the depth information of the user and the user’s hand exists.

In a tabletop holographic display using Azure Kinect, the bottom part of the tabletop is closer than the hand. In addition, several cameras for gaze tracking are attached to the tabletop holographic display, which also causes depth information and interferes with accurate hand detection. Therefore, the depth information of the remaining parts except the moving person has to be erased. Thus, the depth information of the background and the structure is erased using background subtraction [28].

#### 3.2.2. Set ROI

The background and structure were erased with background subtraction. However, a certain amount of noise remains, thus the ROI is set such that the hand can be detected only within the ROI. In the holographic display environment, the position of the camera and the position at which the gesture is made is always constant, thus the ROI is set statically. The mouse pointer is dragged to where the person actually stands in the image to find the coordinates. The ROI is drawn based on these coordinates.

#### 3.2.3. Hand Detection and Bounding Box

Background subtraction erases the depth of the surrounding structures and uses only the depth information of the user to find the user’s hand in the ROI using CrossInfoNet. We trained CrossInfoNet with the NYU hand dataset. As a result, information on 14 joints of the hands, including the center of the palm, can be obtained. The information of each of the 14 joints includes *x*, *y*, and *z* information. After the hand is detected, a bounding box is drawn based on the center of the hand. Bounding box is necessary to visualize the threshold of outputting the up, down, left, right, continuous rotation left and right gestures. The bounding box was drawn as large as the threshold in each of the up, down, left, and right directions, centering on the hand. In addition, the front and back were drawn with the size of 15cm around the hand. Thus, this bounding box was drawn in consideration of the threshold used when recognizing the gesture, and when the finger passes over this bounding box, the corresponding gesture is output.

#### 3.2.4. Gesture Definition and Gesture Recognition

**Figure 7:**
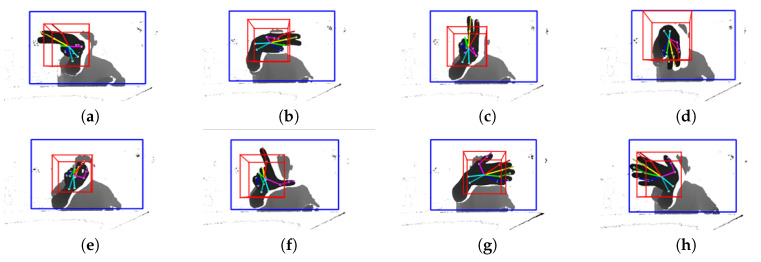
8 hand gestures. (**a**) Left; (**b**) Right; (**c**) Up; (**d**) Down; (**e**) Zoom-out; (**f**) Zoom-in; (**g**) Continuous rotation right; (**h**) Continuous rotation left.

Eight possible gestures can be made: basic rotation up, down, left, right, and zoom in, zoom out, and continuous rotation to the left and right. Figure 7 shows 8 hand gestures. First, the basic rotation is divided into the motion of swiping the index finger and middle finger all the way up, down, left, and right. Intuitively, when swiping in the up, down, left, and right directions, the same gestures as swiping directions are output. Second, the thumb and index finger are used to zoom in and out. The distance between the thumb and forefinger is collected, and the two fingers are spread apart to output the gesture of zooming in. Spreading the thumb and index finger and pinching the two fingers together outputs a zoom out action. Lastly, continuous rotation to the left and right is recognized as a gesture of spreading all fingers and swiping to the left and right.

Each gesture was defined using the relative positional relationship of 14 joints obtained from the result of hand detection, the inner product for each finger, and the distance between fingers. The inner product uses the vector inner product between each finger and the center of the finger and hand. We normalized the value of the inner product from −1 to 1. When a finger is bent, the value of the inner product becomes a negative value close to −1 by the nature of the inner product of the vector, and when the finger is not included, the value of the inner product of the vector approaches 1. This method is used to distinguish gestures based on whether they are bent or stretched for each of the five fingers.

Up, down, left, and right gestures are output when the relative positions of the middle, upper, lower, left, and right joints of the middle finger relative to the center of the hand exceed the threshold, and the inner product of the ring finger and the palm of each finger is negative. The zoom-in and zoom-out gestures use a heap-like arrangement, which continuously stores the distance between the thumb and index finger. When the distance value between the two fingers stacked in the array shows a tendency to increase to exceed the threshold, a zoom in gesture is displayed, and if this distance decreases to exceed the threshold, a zoom out gesture is output. In continuous rotation to the left and right gestures, for all five fingers, the inner product value of the vector with the palm exceeds the threshold, and unlike basic left and right rotation, the ring finger and the little finger are also positioned relative to the palm center. When each threshold is exceeded, a gesture is output. Because the rotation gesture overlaps with the basic left and right gestures and the continuous left and right gestures, it is classified by using the inner product of the vector between the finger and the palm, respectively.

## 4. Experiment Method

**Figure 8:**
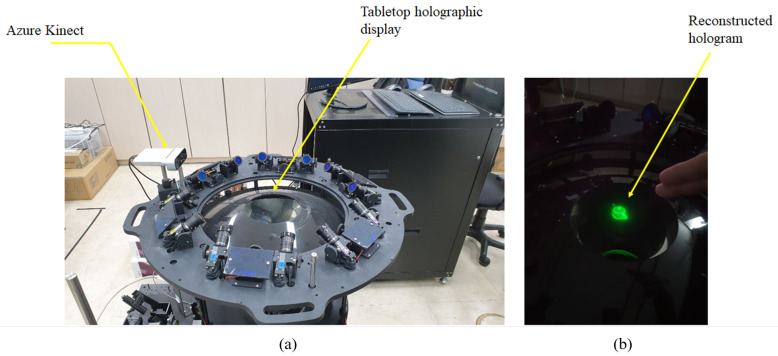
Experiment environment. (**a**) Tabletop holographic environment; (**b**) Gesture interaction environment.

In the experiment, a hologram using only a green laser was used. We conducted experiments at various distances. First, We experimented with 10 people on a total of 8 features we defined. Figure 8a shows the environment of the tabletop holographic display. All the subjects were aware of the gesture operation method and the delay of 1 second and conducted 10 experiments per gesture. All subjects were tested in the same test environment. As shown in Figure 8b, the distance between the camera and the subject’s hand was kept constant at 35–50 cm. The subject performed the experiment in line with the Azure Kinect installed on the tabletop holographic display. Each subject made eight gestures that were tested 80 times. That is, 100 experiments were performed for each gesture. Second, the distance between the camera and the subject’s hand was kept constant at 50–60 cm. Finally, the distance between the camera and the subject’s hand was changed to 60–70 cm.

## 5. Results and Discussions

### 5.1. Results

**Figure 9:**
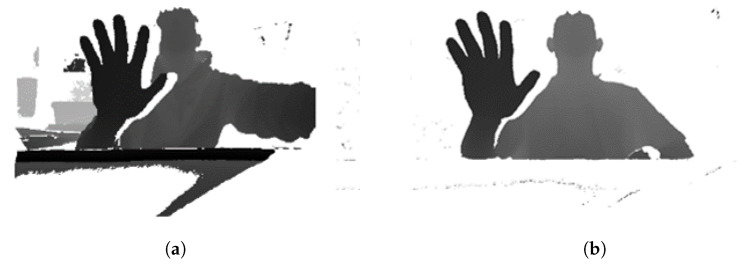
The experimental result before (**a**) and after (**b**) of applying the background subtraction.

Figure 9 shows the result of background subtraction. Figure 9a shows an image that not only includes the depth information of the user but also the structures and backgrounds. As shown in Figure 9b, the depth information of the structure and the background is removed through the background subtraction, and only the depth information of the user is shown.

**Figure 10:**
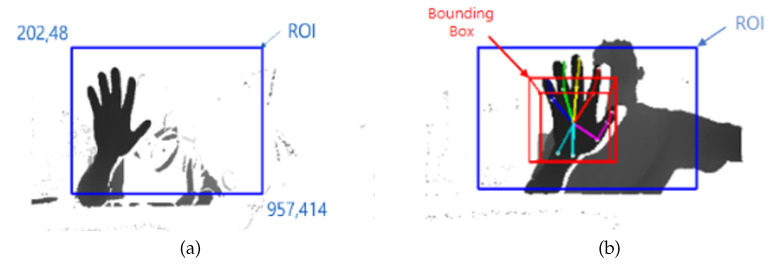
Result of Region of Interest (ROI) and Bounding box. (**a**) Applied ROI; (**b**) Hand detection with 14 joint points and Bounding box.

Figure 10a shows the result of setting the area to find the user’s hand as an ROI. Figure 10b shows a 3D bounding box that shows the result of detecting the hand joint information and the threshold within the ROI.

**Figure 11:**
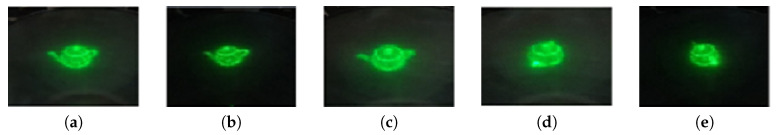
Result of basic rotation gestures. (**a**) Default state; (**b**) Up; (**c**) Down; (**d**) Right; (**e**) Left.

Figure 11 shows the result for the basic rotation gesture. In the default state, if the up gesture is made, the hologram image rotates upward, and when the down gesture is made, the hologram image rotates downward. Gesturing to the right and left has the effect of rotating the hologram image to the right and left, respectively.

**Figure 12:**
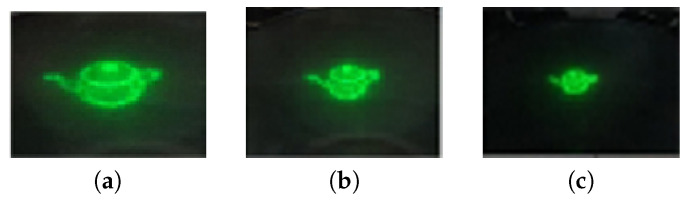
Result of zoom in and out gestures. (**a**) Zoom in; (**b**) Default state; (**c**) Zoom out.

Figure 12 shows the results of the zoom in and zoom out gestures. In the default state, the hologram image becomes larger when the zoom in gesture is detected, and the hologram image becomes smaller when the zoom out gesture is performed.

Table 1 lists the True Positive (TP), False Positive (FP), False Negative (FN), Precision, Recall, and F1 scores [29,30] of each gesture tested 100 times. TP means a condition when a gesture is properly output as a result of performing the gesture. FP means a condition when a gesture is performed, but another gesture is output as a result. FN means a condition when a gesture is performed, but no gesture is output as a result. Table 1 shows the results at a distance of 35–50 cm. For the up gesture, two FN results were obtained, and for the enlarge and reduce gestures, one FP result was obtained. For gesturing to the right with continuous rotation, seven FN results were obtained. The Precision, Recall, and F1 scores were calculated using these results. All the gestures had a precision value of 100, except for the zoom in and zoom out gestures, which yielded a precision value of 99 each. A Recall value of 100 was obtained for all gestures except the right gesture above and continuous rotation right. The Recall values of the up and right hand gestures were 98 and 93, respectively. The F1 score that was calculated using the Precision and Recall values was 98.98 for the up gesture, 99.49 for the zoom in and zoom out gesture, and 96.37 for gesturing to the right with continuous rotation. The F1 score of the remaining gestures was 100.

Table 2 shows the results at a distance of 50–60 cm. For the zoom in and zoom out gestures, Table 2 also obtained 1 false positive result each. False Negative results were obtained for 6 times for the left gesture, 9 times for the right gesture, 2 times for the down gesture, 7 times for the continuous rotation left gesture, and 12 times for the continuous rotation right gesture. All the gestures had a precision value of 100, except for the zoom in and zoom out gestures, which yielded a precision value of 99 each. A Recall value of 100 was obtained for the up gesture, zoom in gesture, zoom out gesture, and continuous rotation left gesture. The Recall value of the left gesture was 94, the right gesture was 91, the down gesture was 98, the continuous rotation left gesture was 93, and the continuous rotation right gesture was 88. The F1 score was 96.90 for the left gesture, 95.28 for the right gesture, 100 for the up gesture, 98.98 for the down gesture, 99.49 for the zoom in and zoom out gesture, 96.37 for the continuous rotation left gesture, and 93.61 for the continuous rotation right gesture.

Table 3 shows the results at a distance of 60–70 cm. For the zoom in and zoom out gestures, Table 3 also obtained 1 false positive result each. False Negative results were obtained for 10 times for the left gesture, 10 times for the right gesture, 9 times for the up gesture, 6 times for the down gesture, 17 times for the continuous rotation left gesture, and 21 times for the continuous rotation right gesture. All the gestures had a precision value of 100, except for the zoom in and zoom out gestures, which yielded a precision value of 99 each. A Recall value of 100 was obtained for the zoom in gesture and zoom out gesture. The Recall value of the left gesture was 90, the right gesture was 90, the up gesture was 91, the down gesture was 94, the continuous rotation left gesture was 83, and the continuous rotation right gesture was 79. The F1 score was 94.73 for the left gesture, 94.73 for the right gesture, 95.28 for the up gesture, 96.90 for the down gesture, 99.49 for the zoom in and zoom out gesture, 90.71 for the continuous rotation left gesture, and 88.26 for the continuous rotation right gesture.

Table 4 combines the results of all gestures of 10 subjects at 35–50 cm, 50–60 cm, and 60–70 cm. The combined results of all the gestures at 35–50 cm were: Precision was 0.99747, Recall was 0.98872, and the F1 score was 0.99307. The combined results of all the gestures at 50–60 cm were: Precision was 0.99747, Recall was 0.95500, and the F1 score was 0.97577. The combined results of all the gestures at 60–70 cm were: Precision was 0.99747, Recall was 0.90875, and the F1 score was 0.95104.

### 5.2. Discussions

Table 1, Table 2 and Table 3 show one false positive result for each of the zoom in and zoom out gestures. The reason for the false positive is the occurrence of a delay of 1 second each time a gesture is output, because the duration of the gesture is shorter than the delay. In Table 1, nine gestures remained undetected, including two up gestures and seven gestures to the continuous rotation right. The false positive result among the up gestures and also among the continuous rotation to the right occurred because the threshold is not exceeded. The hand of an inexperienced subject was smaller than that of the other subjects. In particular, the hand movement in the case of the continuous rotation to the right gesture was more unnatural than the other gestures; thus, the subject with small hands was not able to easily exceed the specified threshold.

Table 2 and Table 3 were tested at a greater distance than Table 1. The amount of the undetected increases as the distance increases. As shown in Table 4, The best results were obtained at 35–50 cm. Especially, the undetected result of a person with a small hand increased significantly. In most ordinary hand-sized people, the amount of the undetected did not increase significantly until the distance between the camera and the user was 70 cm. The experiment was conducted at 70 cm or more, but the hand was not accurately detected and almost no gesture was output. In addition, in the case of a subject whose wrist movement range was not normal, it was difficult to perform the right gesture and the continuous rotation right gesture. Because of the difficulty of performing the right gesture and the continuous rotation right gesture, false negative results often occurred.

If the user and the camera are not in a straight line or are turned more than 30 degrees, the system cannot properly detect the hand. Furthermore, when the camera is rotated more than 30 degrees, the shape of the hand visible on the camera no longer looks like a hand. In addition, only 10 subjects participated in the experiment, thus personal characteristics can influence the outcome. This can be solved by increasing the number of subjects to obtain more objective results.

The field of gesture interaction using Azure Kinect is expanding, such as gesture interaction for drivers in the automotive field and gesture interaction in home appliances by using Azure Kinect. Unlike the approach of these studies, this study developed gesture interaction using only depth information in situations where no light is available. This is a gesture interaction that is more robust to environmental changes than other approaches. If the above problems are solved, gesture interaction using only depth information can be applied to the smart home and automobile fields.

## 6. Conclusions

In this study, we designed a gesture interaction system that uses Azure Kinect to enable the hologram displayed on the tabletop holographic display to be controlled in real time without any equipment. Because the tabletop holographic display requires complete darkness, only depth information is available. Thus, we used Azure Kinect to implement a gesture interaction system that provides high-performance depth information and defined intuitive and easy gestures to render the system user friendly. As a result, precision and recall values of 0.99747 and 0.98872 were obtained, respectively, and finally, an excellent F1 score of 0.99307 was achieved. However, people with small hands could encounter the problem of undetectableness. The false positive rate caused by this problem could be reduced by allowing the threshold to change flexibly by considering the size of the user’s hand. In the future, we plan to improve the system to allow users with small hands to use it without any prior exploration. In addition, through experiments, we found that people’s right wrist bending behavior was more difficult than others. Therefore, it was assumed that it was necessary to lower the threshold of the gesture to twist the wrist to the right compared to other gestures. This system enables the user to control the hologram of the tabletop holographic display without using other equipment. As the demand for holograms increases and the amount of research in this field increases, the implementation of a larger number of gestures in the future would enable the user to control the hologram more freely with their own hands without requiring additional equipment.

## Figures and Tables

**Table 1:** Experiment results of precision error, recall error, and F1 score about each gesture of 10 subject at 35–50 cm.

Gesture	Left	Right	Up	Down	Zoom-In	Zoom-Out	Continue Left	Continue Right
Total Attempts	100	100	100	100	100	100	100	100
True Positive	100	100	98	100	99	99	100	93
False Positive	0	0	0	0	1	1	0	0
False Negative	0	0	2	0	0	0	0	7
Precision	100	100	100	100	99	99	100	100
Recall	100	100	98	100	100	100	100	93
F1 score	100	100	98.98	100	99.49	99.49	100	96.37

**Table 2:** Experiment results of precision error, recall error, and F1 score about each gesture of 10 subjects at 50–60 cm.

Gesture	Left	Right	Up	Down	Zoom-in	Zoom-out	Continue Left	Continue Right
Total Attempts	100	100	100	100	100	100	100	100
True Positive	94	91	100	98	99	99	93	88
False Positive	0	0	0	0	1	1	0	0
False Negative	6	9	0	2	0	0	7	12
Precision	100	100	100	100	99	99	100	100
Recall	94	91	100	98	100	100	93	88
F1 score	96.90	95.28	100	98.98	99.49	99.49	96.37	93.61

**Table 3:** Experiment results of precision error, recall error, and F1 score about each gesture of 10 subjects at 60–70 cm.

Gesture	Left	Right	Up	Down	Zoom-In	Zoom-Out	Continue Left	Continue Right
Total Attempts	100	100	100	100	100	100	100	100
True Positive	90	90	91	94	99	99	83	79
False Positive	0	0	0	0	1	1	0	0
False Negative	10	10	9	6	0	0	17	21
Precision	100	100	100	100	99	99	100	100
Recall	90	90	91	94	100	100	83	79
F1 score	94.73	94.73	95.28	96.90	99.49	99.49	90.71	88.26

**Table 4:** Experiment results of total precision error, total recall error, and total F1 score.

Distance	35–50 cm	50–60 cm	60–70 cm
Precision	0.99747	0.99747	0.99747
Recall	0.98872	0.95500	0.90875
F1 score	0.99307	0.97577	0.95104

## References

[1] Ren Z., Yuan J., Meng J., Zhang Z. (2013). Robust part-based hand gesture recognition using kinect sensor. IEEE Trans. Multimed..

[2] Biswas K.K., Basu S.K. Gesture recognition using microsoft kinect. In Proceeding of the 5th International Conference on Automation, Robotics and Applications, James Cook Hotel.

[3] Li Y. Hand gesture recognition using Kinect. In Proceeding of the 2012 IEEE International Conference on Computer Science and Automation Engineering.

[4] Marin G., Dominio F., Zanuttigh P. Hand gesture recognition with leap motion and kinect devices. In Proceeding of the 2014 IEEE International Conference on Image Processing.

[5] Patsadu O., Nukoolkit C., Watanapa B. Human gesture recognition using Kinect camera. In Proceeding of the 2012 Ninth International Conference on Computer Science and Software Engineering.

[6] Guzsvinecz T., Szucs V., Sik-Lanyi C. (2019). Suitability of the Kinect sensor and Leap Motion controller—A literature review. Sensors.

[7] He G.F., Kang S.K., Song W.C., Jung S.T. Real-time gesture recognition using 3D depth camera. In Proceeding of the 2011 IEEE 2nd International Conference on Software Engineering and Service Science.

[8] Ito A., Nakada K. UI Design based on Traditional Japanese Gesture. In Proceeding of the 2019 10th IEEE International Conference on Cognitive Infocommunications.

[9] Ferri J., Llinares Llopis R., Moreno J., Ibañez Civera J., Garcia-Breijo E. (2019). A wearable textile 3D gesture recognition sensor based on screen-printing technology. Sensors.

[10] Zsolczay R., Brown R., Maire F., Turkay S. Vague gesture control: Implications for burns patients. In Proceeding of the 31st Australian Conference on Human-Computer-Interaction.

[11] Jiang L., Xia M., Liu X., Bai F. (2020). Givs: Fine-Grained Gesture Control for Mobile Devices in Driving Environments. IEEE Access.

[12] Streeter L., Gauch J. Detecting Gestures Through a Gesture-Based Interface to Teach Introductory Programming Concepts. In Proceeding of the International Conference on Human-Computer Interaction.

[13] Bakken J.P., Varidireddy N., Uskov V.L. Smart Universities: Gesture Recognition Systems for College Students with Disabilities. In Proceeding of the 7th International KES Conference on Smart Education and e-Learning.

[14] Kim J., Lim Y., Hong K., Kim H., Kim H.E., Nam J., Park J., Hahn J., Kim Y.J. (2019). Electronic tabletop holographic display: Design, implementation, and evaluation. Appl. Sci..

[15] Lim Y., Hong K., Kim H.E., Chang E.Y., Lee S., Kim T., Nam J., Choo H.G., Kim J., Hahn J. (2016). 360-degree tabletop electronic holographic display. Opt. Express.

[16] Chang E.Y., Choi J., Lee S., Kwon S., Yoo J., Park M., Kim J. (2018). 360-degree color hologram generation for real 3D objects. Appl. Opt..

[17] Nguyen X.S., Brun L., Lézoray O., Bougleux S. A neural network based on SPD manifold learning for skeleton-based hand gesture recognition. Proceedings of the IEEE Conference on Computer Vision and Pattern Recognition.

[18] Wan C., Probst T., Gool L.V., Yao A. Self-supervised 3d hand pose estimation through training by fitting. Proceedings of the IEEE Conference on Computer Vision and Pattern Recognition.

[19] Du K., Lin X., Sun Y., Ma X. Crossinfonet: Multi-task information sharing based hand pose estimation. Proceedings of the 2019 IEEE Conference on Computer Vision and Pattern Recognition.

[20] Wang C., Liu Z., Chan S.C. (2014). Superpixel-based hand gesture recognition with kinect depth camera. IEEE Trans. Multimed..

[21] Supancic J.S., Rogez G., Yang Y., Shotton J., Ramanan D. Depth-based hand pose estimation: Data, methods, and challenges. Proceedings of the IEEE International Conference on Computer Vision.

[22] Joo S.I., Weon S.H., Choi H.I. (2014). Real-time depth-based hand detection and tracking. Sci. World J..

[23] Liu X., Fujimura K. Hand gesture recognition using depth data. Proceedings of the Sixth IEEE International Conference on Automatic Face and Gesture Recognition.

[24] Poularakis S., Katsavounidis I. Finger detection and hand posture recognition based on depth information. Proceedings of the 2014 IEEE International Conference on Acoustics, Speech and Signal Processing.

[25] Ren Z., Meng J., Yuan J. Depth camera based hand gesture recognition and its applications in human-computer-interaction. Proceedings of the 2011 8th International Conference on Information, Communications Signal Processing.

[26] Yu M., Kim N., Jung Y., Lee S. (2020). A Frame Detection Method for Real-Time Hand Gesture Recognition Systems Using CW-Radar. Sensors.

[27] Abavisani M., Joze H.R.V., Patel V.M. Improving the performance of unimodal dynamic hand-gesture recognition with multimodal training. Proceedings of the IEEE Conference on Computer Vision and Pattern Recognition.

[28] Lee S.H., Lee G.C., Yoo J., Kwon S. (2019). Wisenetmd: Motion detection using dynamic background region analysis. Symmetry.

[29] Yin Y., Randall D. Gesture spotting and recognition using salience detection and concatenated hidden markov models. Proceedings of the 15th ACM on International conference on multimodal interaction, Coogee Bay Hotel.

[30] Kim J.H., Hong G.S., Kim B.G., Dogra D.P. (2018). deepGesture: Deep learning-based gesture recognition scheme using motion sensors. Displays.

